# Dental procedures cause stress in children with cerebral palsy?

**DOI:** 10.4317/jced.58392

**Published:** 2021-11-01

**Authors:** Maria de Fátima-Monteiro Tomasin, Giselle-Rodrigues de Sant’Anna, Adriano-Tomio Hoshi, Danilo-Antônio Duarte

**Affiliations:** 1 Department of Pediatric Dentistry, School of Dentistry, Western Paraná State University, Brazil; 2 Department of Graduate Studies, São Leopoldo Mandic School of Dentistry, Brazil

## Abstract

**Background:**

To evaluate the level of stress during dental care in children and preadolescents with cerebral palsy through biological and psychological parameters.

**Material and Methods:**

A total of 38 children aged 7 to 12 years were divided into two groups: one with 18 children with cerebral palsy and the other with 20 healthy children (control group). Saliva was noninvasively collected before and after dental care to analyze salivary biomarkers. The Frankl Behavior Scale and the Facial Image Scale were applied.

**Results:**

After the dental procedure, cortisol levels were significantly higher (*p* = 0.02) in the cerebral palsy group than in the control group. Salivary alpha-amylase was not significantly different between groups. Regarding psychological parameters, anxiety was significantly higher (*p* = 0.00012) in the cerebral palsy group than in the control group.

**Conclusions:**

There was a change in physiological parameters (salivary cortisol and salivary alpha-amylase) and in psychological parameters (Frankl Behavioral Scale and Facial Image Scale) in patients with cerebral palsy, who exhibited higher stress and anxiety levels than did children without cerebral palsy.

** Key words:**Ortisol, physiological stress, dental care, cerebral palsy, dental treatment anxiety.

## Introduction

The classic concept of disability seen as a medical model has been replaced by a social model concept. This means recognizing that disability is the product of individuals with disabilities and behavioral and environmental barriers that limit or prevent equal participation in interpersonal and social activities (1). Individuals with cerebral palsy (CP) are certainly included in this concept. CP is described as a neurodevelopmental disorder responsible for physical and cognitive impairment, with a prevalence of 2-2.5 cases per 1000 live births. It is considered the most common cause of physical disability in childhood (2). Children with CP require special medical and dental care because caries, periodontal diseases and malocclusions are relatively frequent (3-5), caused by difficulties in oral hygiene, dietary consistency, drugs, misinformation and limited access to dental services (6). Dental procedures usually are laborious due to the involuntary movements, constant spasms, marked stiffness and communication difficulties (2,7). In this line of reasoning, it is ineviTable that children with CP will require potentially anxiety- and stress-provoking dental interventions (3). However, there are few studies on this topic for this group of patients (8). The knowledge about the level of stress and anxiety can contribute to a better preparation of dental surgeon and his team in the proper care of these patients. Considering that salivary secretion may be affected by stressors, the present study chose the biomarkers cortisol and salivary alpha-amylase (sAA) (9,10) and used the Frankl Behavior Scale (FBS) (11) and Facial Image Scale (FIS) (12), classical instruments used to analyze the emotional state of children, to assess the level of stress in children and preadolescents with CP.

## Material and Methods

This was a cross-sectional study, conducted from 03/2019 to 11/2019, and the study subjects were children treated at the Pediatric Dentistry Clinic and Center of Dental Specialties. This study was approved by the research ethics committee of São Leopoldo Mandic School of Dentistry under number 2.431.543/2017, meeting the ethical and scientific requirements of Resolution 466/2012 of the National Health Council (Norms for Research involving Human Beings).

-Sample Size Calculation and Selection 

G Power 3.1 software (University of Dusseldorf) was used to calculate the sample size required to detect an effect size of 0.8, using the t-test for independent samples (cortisol), with an alpha error of 0.05 and 80% test power; the calculated sample size was 42 research subjects. A total of 42 individuals of both sexes and aged 7 to 12 years were randomly selected. The participants signed an informed assent form, and their guardians signed an informed consent form. Two groups were formed at a 1:1 ratio, defined as the cerebral palsy group (CPG) and the control group (CG). Individuals with CP were included in the CPG, and individuals without CP were included in the CG. Children with severe intellectual disabilities who used corticosteroids or drugs that altered oxygen saturation were excluded.

-Clinical Procedures and Saliva Collection 

Saliva was collected in the evening to minimize the effects of the circadian cycle. A specific sampling device was used for collection (Salivette, Sarstedt), i.e., a centrifuge tube containing a cotton swab. With each individual in a dental chair, the cotton swab was placed on the floor of the mouth until completely moistened with saliva, and then the swab was returned to the tube. Saliva was collected from the participants in duplicate, before and after the dental prophylaxis. Both the saliva collection and the clinical procedure were performed by the researchers, who have expertise in pediatric dentistry and special needs patients.

**Figure 1:**
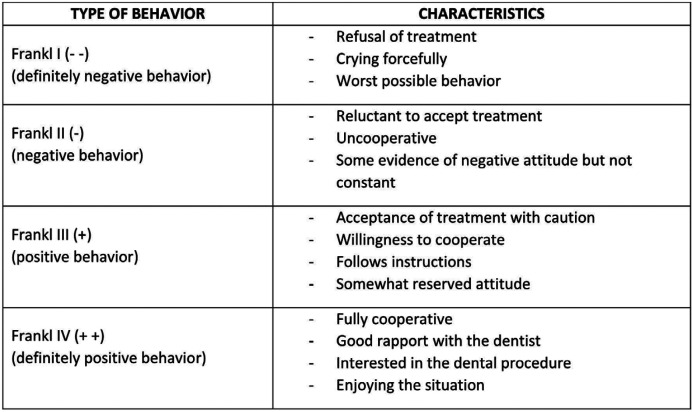
Frankl Behavior Scale.

**Figure 2:**
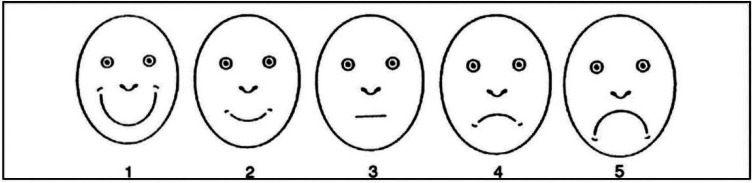
Facial Image Scale.

For the analysis of cortisol, tubes containing saliva were sent under refrigeration (2º to 8ºC) to UNILABOR in Cascavel, Paraná. The tubes were centrifuged at 1000 g to isolate the saliva, which was then subjected to electrochemiluminescence; the results are expressed in nmol/L. The tubes for the analysis of sAA were sent to the Biochemistry Laboratory in an ice box. The tubes were centrifuged at 4,500 rpm for 10 minutes, stored at -20ºC and subsequently analyzed. The analysis was performed using the method described by Fisher and Stein (13). Briefly, maltose was used as a standard, with the sample incubated in 1% starch solution in 20 mM phosphate buffer, pH 7.0, for 5 minutes at 37ºC. The reaction was stopped by adding dinitrosalicylic acid, and the mixture was kept in water at 98ºC for 5 minutes. The sample was read in a spectrophotometer at 540 nm, and the results are expressed in U/ml. For the evaluation of behavior during care, a trained pediatric dentist who was not a participant in the study applied the Frankl Behavior Scale (11) (FBS), an observational scale that classifies behavior into four categories (Fig. 1). The FIS (12) comprises a series of five face Figures, ranging from very unhappy (5) to very happy (1) (Fig. 2). The scale was presented to each individual by the researcher after the procedure so that he or she could choose the face that corresponded to their feelings during the dental procedure. For statistical analysis, the following tests were used: Student’s t-test for paired samples, Yuen’s t-test and non-parametric Mann-Whitney test for independent samples. The data were analyzed using the statistical software R for Windows, version 3.6.2 (2019-12-12). A 5% significance level was considered for all analysis.

## Results

After the necessary exclusions, the sample included 38 children and preadolescents (22 females and 16 males), with 20 participants allocated to the CG and 18 to the CPG (Table 1). Table 2 shows the cortisol and sAA values in the CG and CPG. Regarding cortisol, the mean and standard deviation could not be used as measures of central tendency due to asymmetry on the right; therefore, the median and interquartile range (IQR) were used instead. The CPG showed greater data variability, evidenced by the IQR at both evaluation times. Based on the medians, the effect of the intervention on the groups was opposite, with a reduction in cortisol levels in the CG and an increase in the CPG. There was a significant difference only in the comparison between groups after the intervention (*p* = 0.02). Regarding sAA, the results showed no significant difference between groups. Table 3 shows the results of the FBS and FIS evaluations. For the FBS, positive scale ratings were the most frequent results in both groups. For the FIS, the CG showed a predominance of level 1 (very happy), while for the CPG, there was a more dispersed distribution, with a predominance of level 4 (unhappy). There was a significant difference (*p* = 0.00012) in the scores for both scales between the groups.

**Table 1:**
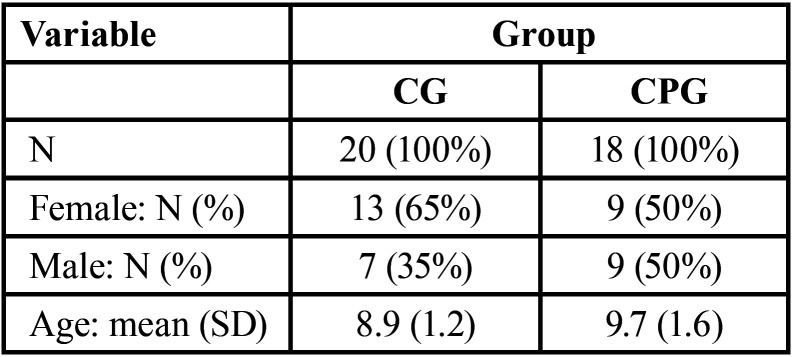
Sample characteristics according to group (CG and CPG). The data are presented as absolute values with percentages in parentheses. For age, the data are presented as the mean and standard deviation (SD).

**Table 2:**
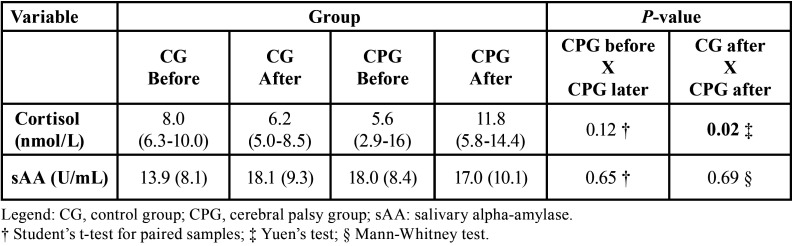
Cortisol and salivary alpha-amylase levels, as well as their respective p values, in the CG and CPG. The data are presented as the mean, with the standard deviation in parentheses, or as the median and interquartile range, when two numbers are presented in parentheses.

**Table 3:**
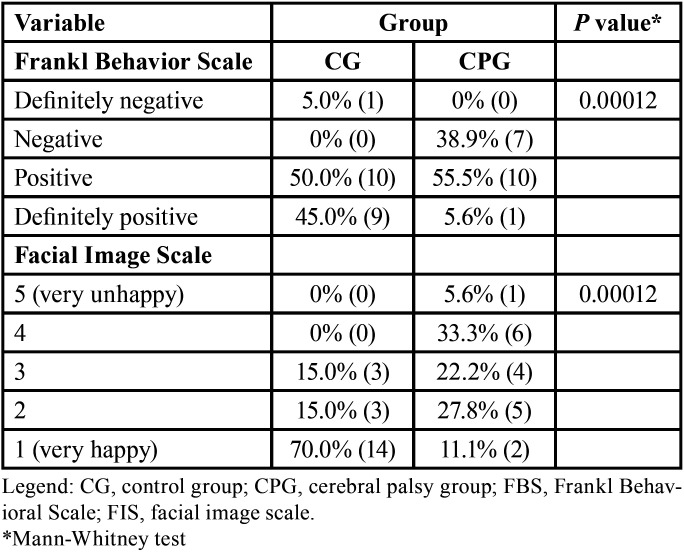
Distribution of data (percentage and count), with p values, obtained using behavioral scales (FBS and FIS) after the intervention.

## Discussion

It is recognized that children with CP have a high rate of dental treatment needs (3,4). Considering the exponential demand for dental care in this population (14), it is necessary to understand the level of stress experienced during clinical procedures. It is noteworthy that anxiety is a limiting factor in clinical interventions in children (15) and is amplified in those with CP, the target population of our study. To evaluate stress levels using physiological parameters, we chose salivary cortisol and sAA because samples for their analysis can be collected using noninvasive and painless procedures (16,17). Painful stimuli produce a physiological response through excitation of the hypothalamic-pituitary-adrenal axis, resulting in the release of cortisol. sAA is an important salivary enzyme and is used as a potential marker of stress responses via central nervous system activity (9,18).

Considering behavioral and subjective measures, we chose to use the FBS and FIS to assess anxiety. Both instruments are validated, have established application, are easy to apply and have high reliability (12,19,20). The use of a physiological indicator combined with a psychological indicator contributed to the reliability of our results. Cortisol levels significantly increased in the CPG after dental care; in the CG, there was a moderate decrease in cortisol. Although sAA showed a slight decrease from pretreatment to posttreatment in the CPG, the difference was not significant. The difference between cortisol and sAA can be explained by the fact that cortisol is more sensitive to physical stimuli and that sAA is more sensitive to psychological stressors (21,22). Due to the scarcity of studies in the literature on the level of stress in children with CP, the other studies used for comparison with our study are different. Blomqvist *et al*. (23) and dos Santos *et al*. (18) analyzed populations with attention deficit/hyperactivity disorder (ADHD) and global developmental delay (GDD), respectively, and found no significant differences in salivary cortisol levels between these groups and the controls. They also did not observe significant differences in cortisol levels before and after dental examinations. In contrast to these studies, others have observed higher salivary cortisol levels before dental prophylaxis, which may indicate that the anticipation of a dental visit generates greater anxiety than the visit itself (10,22,24). However, in the present study, we observed a significant difference (*p* = 0.02) in cortisol levels between groups after treatment. In the CPG, cortisol levels were higher after the intervention, similar to the findings of Yfanti *et al*. (25) and Gomes *et al*. (26), who analyzed healthy children. In the analysis of sAA, we observed in our study that there were no significant changes in sAA levels before and after dental treatment, both in the CPG and in the CG, similar to the findings of Yfanti *et al*. (25). There is a likely explanation for this result: the UNIOESTE Dental Clinic is a reference for care; therefore, the children and preadolescents in our study had previous experience with dental procedures.

In contrast, other studies have shown significantly higher sAA levels before dental procedures (18,25). Furthermore, Rodrigues Gomes *et al*. (22) observed higher sAA levels after treatment. In addition to the disability, other factors can influence sAA responses, such as previous invasive dental procedures and age, as children in the age group below 72 months have higher sAA levels on clinical examination (15). Regarding the scales, our results showed that for the CG, the predominant FIS score was 1 (very happy), and for the CPG, the predominant score was 4 (unhappy); the difference in scores was significant (*p*<0,01). Other studies also found that the predominant FIS score was 1 in healthy children (27,28). Regarding the FBS, some studies have reported that most children showed positive or definitely positive behavior during dental care, corroborating our findings in the CG (95%) (18,22,29). However, 38.9% of the CPG exhibited negative behavior, demonstrating a significant difference (*p*<0,01) between the CG and CPG groups. Other studies found that predominant FIS scores were 2 and 3 in healthy children (27,28).

In a study by dos Santos (18) children with global developmental delay, no children showing true negative and 60,6% positive behavior based on the FBS. It is important to note that several factors, such as pain and age, can cause more negative behavior because younger patients, in addition to being more immature and susceptible to fear, are usually more agitated, requiring greater agility in care and knowledge of behavioral management techniques (30). In patients with CP, behavior may also be influenced by factors such as the level of neurological involvement, involuntary movements and difficulty in opening the mouth (2).

Our postclinical intervention results indicate that the physiological and psychological parameters confirm the behavior of the study population, especially those in the CPG, who had higher cortisol levels, a higher occurrence of negative behaviors, based on the FBS, and a higher occurrence of anxiety, based on the FIS. These results indicate that patients with CP presented greater anxiety and stress regarding dental treatment. It is important to highlight some limitations of this study, such as greater sampling difficulty, verbal inability of the study population, and the occasional need for physical restraint. The comparison between the groups may be questionable, but the inclusion of healthy children was necessary due to the use of physiological markers as an objective parameter. In turn, these limitations were compensated by the methodological care in data collection, minimizing information bias. Despite the limitations and the small sample of this study, the results reinforce the analysis of the physiological parameters cortisol and sAA and of the Frankl Behavior and Facial Image scales indicated that dental procedures produced anxiety and stress in both groups, notably to a greater degree in the CPG. In addition, the results indicate that stress and anxiety produced during dental procedures should be managed by the dental surgeon. For this, it is necessary to invest in professional training policies starting in undergraduate curricula and extending to continuing education. These actions would certainly contribute to alleviating anxiety and stress and are essential for promoting the health of this population, which is sometimes underserved.
